# Correlation between Body Mass Index and Waist Circumference in Patients with Metabolic Syndrome

**DOI:** 10.1155/2014/514589

**Published:** 2014-03-04

**Authors:** Marcin Gierach, Joanna Gierach, Marlena Ewertowska, Adam Arndt, Roman Junik

**Affiliations:** ^1^Department of Endocrinology and Diabetology and Laboratory of Nuclear Medicine, Nicolaus Copernicus University in Torun, Collegium Medicum in Bydgoszcz, 85-094 Bydgoszcz, Poland; ^2^Internal Ward in District Hospital in Wabrzezno, 87-200 Wabrzezno, Poland

## Abstract

Metabolic syndrome is defined as a group of coexisting metabolic risk factors, such as central obesity, lipid disorders, carbohydrate disorders, and arterial hypertension. According to the 2005 IDF criteria, subsequently revised in 2009, abdominal obesity is identified as the waist circumference of ≥80 cm in women and ≥94 cm in men. It is responsible for the development of insulin resistance. The aim of our study was to demonstrate a correlation between waist circumference (WC) and body mass index (BMI) in patients with metabolic syndrome in relation with hypertension, lipid disorders, and carbohydrate disorders. A cross-sectional two-site study was conducted in the Kuyavian-Pomeranian Voivodeship for 24 months. The study group consisted of 839 patients with diagnosed metabolic syndrome: 345 men (41.1%) and 494 women (58.9%) aged 32–80. In the study group, WC was found to be significantly correlated with BMI (*R* = 0.78, *P* < 0.01). The presence of overweight in men (BMI 25, 84 kg/m^2^) and even normal body weight in women (BMI 21,62 kg/m^2^) corresponds to an increased volume of visceral tissue in the abdomen. Introduction of primary prophylaxis in those people to limit the development of diabetes mellitus type 2 and cardiovascular diseases should be considered.

## 1. Introduction

Metabolic syndrome is defined as a group of coexisting metabolic risk factors, such as central obesity, lipid disorders, carbohydrate disorders, and arterial hypertension [1–5]. Those factors increase the risk of developing cardiovascular diseases of atherosclerotic etiology and diabetes mellitus type 2 [1, 5–9], which are the main cause of premature deaths among most of the European and US population [10].

The number of patients with metabolic syndrome increases with age. In the US population, the percentage of such patients above the age of 20 is approximately 23%, while the percentage of such patients above 60 is approximately 40% [11].

Abdominal obesity is the major disorder constituting a base for the development of metabolic syndrome. BMI is the simplest, most practical, and most widely used system of indexing body weight. It is defined as body weight (in kilograms) divided by the square of body height (in metres). The index divides patients into appropriate categories: underweight, normal weight, overweight, and obese. Even though BMI is commonly used for monitoring the occurrence of obesity in the population, it has numerous limitations. It does not provide any information on the distribution of the adipose tissue in the organism. BMI is a calculated statistical value which does not take into consideration physiological differences in the proportions between the adipose, osseous, and muscular tissues [11]. Besides, its value is affected by sex, age, constitution, and training. Evidence from the conducted studies has revealed that abdominal obesity (assessed based on the waist circumference) plays a very important role in the development of metabolic disorders and in the assessment of cardiovascular risk. According to the 2005 IDF criteria, subsequently revised in 2009, abdominal obesity is identified as the waist circumference of ≥80 cm in women and ≥94 cm in men. It is responsible for the development of insulin resistance which decreases the levels of the HDL-cholesterol fraction, increases the levels of triglycerides, and leads to the development of arterial hypertension. All of the above-mentioned disorders contribute to metabolic syndrome and are related to the development of type 2 diabetes and ischaemic heart disease.

Due to the limitations of BMI methodology, current reports by the World Health Organization and other organizations suggest combining the measurements of BMI and abdominal obesity [11]. The conducted studies have revealed that it is necessary to determine the adipose tissue distribution in the organism.

The aim of our study was to demonstrate a correlation between waist circumference (WC) and body mass index (BMI) in patients with metabolic syndrome in relation to hypertension, lipid disorders, such as atherogenic dyslipidaemia, and carbohydrate disorders, such as impaired fasting glucose or diabetes mellitus type 2.

## 2. Materials and Methods

A cross-sectional two-site study was conducted in the Kuyavian-Pomeranian Voivodeship for 24 months. The study group consisted of 839 patients with diagnosed metabolic syndrome: 345 men (41.1%) and 494 women (58.9%) aged 32–80 (Table 1).

Anthropometric measurements (height, weight, and waist circumference) were taken in all subjects. Body mass index (BMI) was calculated as body weight (in kilograms) divided by the square of body height (in metres).

Demographic factors (age, sex, and obesity) and lifestyle factors (smoking habits, physical activity, and alcohol consumption) were determined.

The main analysis was performed using the 2009 revision of the International Diabetes Federation (IDF) definition, requiring the presence of the following criteria:

- abdominal obesity: waist circumference of ≥80 cm in women and ≥94 cm in men, measured at the umbilical level in a standing position;
- systolic blood pressure (SBP) of ≥130 mmHg or diastolic blood pressure (DBP) of ≥85 mmHg (or treatment with an antihypertensive medication), measured in all subjects using a validated digital electronic tensiometer in both upper limbs in a seated position after 15 minutes of rest;
- triglycerides levels (TG) of ≥150 mg/dL (1.7 mmol/L) or statin/fibrate treatment; HDL-cholesterol levels (HDL-C) of <50 mg/dL (1.3 mmol/L) in women and <40 mg/dL (1.0 mmol/L) in men (or statin/fibrate treatment) were measured using an AutoAnalyzer;
- fasting glycaemia of ≥100 mg/dL (5.6 mmol/L) (or treatment with antidiabetic medications), measured in all subjects with central obesity (WC > 80 cm in women and >94 cm in men). Diabetes was defined as fasting blood glucose (FBG) levels of ≥7 mmol/L and/or treatment with antidiabetic medications.

The study protocol was approved by the Ethics Committee at the University Hospital in Bydgoszcz. All subjects granted their informed consent for participation in the study. Study group characteristics are presented in Table 1.

Metabolic syndrome was identified based on 3 traits in 290 (34.6%) patients, 4 traits in 392 (46.7%) patients, and 5 traits in 157 (18.7%) patients. The characteristics of the study group according to each component of metabolic syndrome are presented in Table 2.

All statistical analyses were performed using the STATISTICA 10.0 software (StatSoft Poland, Bydgoszcz). The Mann-Whitney test, a nonparametric version of Student's *t*-test for independent variables, was used for the comparison. No parametric tests were used in the analyses as for some variables the assumptions of Student's *t*-test regarding normal distribution and variance were not met. The value of Pearson's linear correlation coefficient was determined in the analysis. The results were considered as statistically significant at *P* < 0.05. Bilateral test was used to determine the significance of the differences between correlation coefficients.

## 3. Results

In the study group, waist circumference was found to be significantly correlated with BMI (*R* = 0.78, *P* < 0.01). The correlation was more pronounced among women (*r* = 0.80) than among men (*r* = 0.76) (Table 3).

Correlation between BMI and waist circumference was analysed according to each component of metabolic syndrome.

### 3.1. Groups with Arterial Hypertension and Normal Arterial Blood Pressure

A group of patients with arterial hypertension was compared with a group of patients with normal values of arterial blood pressure. It was determined that in the group with arterial hypertension (796; 94.9% analysed patients), the correlation coefficient (*r* = 0.79, *P* < 0.05) was very similar to that of the entire study group. Conversely, in 43 patients with normal arterial blood pressure the correlation was less pronounced (*r* = 0.63, *P* < 0.05). The difference between the two groups was statistically significant (0.79 versus 0.63, *P* = 0.04).  Table 4 presents the statistically significant differences between the group of patients with arterial hypertension and the group of patients with normal arterial blood pressure.

Table 5 presents the correlation between BMI and waist circumference according to the presence of arterial hypertension or normotension.

Subsequently, the study group was divided according to subject sex. It was determined that in the subgroup of men with normal arterial blood pressure, the correlation between BMI and WC (0.67; *P* < 0.05) was more pronounced than that observed in the subgroup of women with normotension (0.55; *P* < 0.05). However, the difference was not statistically significant (0.55 versus 0.67 *P* = 0.5632).

### 3.2. Groups with Diabetes Mellitus Type 2 and without Carbohydrate Disorders

Correlation between BMI and WC in the study group with type 2 diabetes, compared to the group without carbohydrate disorders, is presented in Table 6.

Type 2 diabetes was determined in 491 (58.5%) patients with metabolic syndrome, while in 110 (13.5%) patients no carbohydrate disorders were found (Table 7).

### 3.3. Groups of Patients with and without Impaired Fasting Glucose (IFG)

In 208 (24.8%) patients, impaired fasting glucose levels were detected (Table 9).

### 3.4. Groups of Patients with Decreased Levels of the HDL-Cholesterol Fraction (<50 in Women and <40 in Men) and without Lipid Disorders

In 567 (67.6%) patients, decreased levels of the HDL-cholesterol fraction were detected (Tables 10 and 11).

### 3.5. Groups of Patients with Increased Levels of Triglycerides (>150 mg/dL) and Normal TG Levels

In 313 (37.3%) patients, increased levels of triglycerides (TG) were detected, as opposed to 526 (62.7%) patients in whom increased TG levels were not observed (Tables 12 and 13).

## 4. Discussion

Metabolic syndrome (MetS) is a serious public health problem worldwide and its occurrence is increasing [1, 2, 10]. In population studies, the detected MetS occurrence varies between 20% and 45% of population. This constitutes a serious social problem as in such patients the risk of cardiovascular diseases increases significantly.

Abdominal obesity is the most frequently observed component of metabolic syndrome. In the NATPOL 2002 study, it was detected in 19% patients, while in the 2011 NATPOL PLUS study, the percentage increased to 22% of Polish population. Currently, it is estimated that abdominal obesity is present in approximately 6.5 million residents of Poland, while in the predictions for the year 2035 the value is expected to increase to 10 million.

Obesity is commonly assessed using body mass index (BMI) whose values of ≥30 kg/m^2^ determine somatic obesity.

In our study, we compared the correlation between BMI and waist circumference in patients with diagnosed metabolic syndrome (Table 8). We distinguished the components of metabolic syndrome according to subject sex. We determined that the correlation between BMI and WC occurred in each of the studied subgroups. The correlation coefficient is the lowest (*r* = 0.55) in the subgroup of women with diagnosed metabolic syndrome including arterial hypertension. This is probably due to the small size of the subgroup (*n* = 20) and lower BMI and WC values versus the subgroup with arterial hypertension (BMI 28.4 versus 30.8, resp., and WC 104.6 versus 108.0, resp.).

The results obtained in the study group for both women and men with BMI = 30 kg/m^2^ correlate with waist circumference of 104 cm in women and 108 cm in men. According to the IDF criteria 2009 revision, abdominal obesity in the European population is defined as WC of ≥80 cm in women and WC of ≥94 cm in men. The above-mentioned values correspond to BMI = 21,62 kg/m^2^ in women and BMI = 25,84 kg/m^2^ in men, respectively. The former value is normal, while in the latter case only the “overweight” criterion is met. A question to consider is whether a revision of the current BMI-based obesity criteria should be made or whether using those criteria in the assessment of abdominal obesity should be abandoned. Aye and Sazali suggest that WC is a better predictor of metabolic risk factors for developing MetS than BMI and propose that metabolic risk factors should be screened in the measured WC value in both genders exceeding 80 cm, regardless of BMI [6]. Similarly, Bouguerra et al. conclude that waist circumference (WC) is a convenient measure of abdominal adipose tissue, which is a diabetes risk factor and is strongly linked to other cardiovascular disease (CVD) risk factors [12]. Other authors conducting studies on other ethnic groups also suggest introducing certain changes regarding the use of BMI. Ko and Tang propose the creation of an intermediate state of high WC, the “central preobesity” for Chinese men with WC of 84–90 cm and women with WC of 74–80 cm. People with central preobesity, similar to those with overweight (BMI 23–25 kg/m^2^), already have an increased risk of comorbidities [13]. On the other hand, a number of studies have also shown that BMI is as effective as waist circumference in predicting the development of type 2 DM and other metabolic disturbances [14–17]. In addition, the Japan Society for the Study of Obesity has reported that BMI may estimate visceral fat measured using computed tomography as robustly as waist circumference and that obesity-related complications increase with BMI values of 25 [1, 18].

## 5. Conclusions

1. There is a strong linear correlation between waist circumference and BMI values.
2. The presence of overweight in men (BMI 25,84 kg/m^2^) and even normal body weight in women (BMI 21,62 kg/m^2^) corresponds to an increased volume of visceral tissue in the abdomen. Introduction of primary prophylaxis in those people to limit the development of diabetes mellitus type 2 and cardiovascular diseases should be considered.

## Figures and Tables

**Table 1:** General characteristics of the study group.

	*n*	Age (years)	Body weight (kg)	Body height (cm)	BMI (kg/m^2^)	Waist circumference (cm)
Total	839	67.5 ± 12.9	84.9 ± 17.8	166.0 ± 8.6	30.7 ± 5.6	107.9 ± 13.5
Women	494(58.9%)	70.6 ± 11.3	79.5 ± 17.5	161.1 ± 5.8	30.6 ± 6.3	105.8 ± 14.6
Men	345(41.1%)	63.0 ± 13.8	92.5 ± 15.1	173.1 ± 6.7	30.8 ± 4.4	110.8 ± 11.2

**Table 2:** Characteristics of the study group according to the components of metabolic syndrome.

	*n*	Age (years)	BMI (kg/m^2^)	Waist circumference (cm)
Total	839	67.5 ± 12.9	30.7 ± 5.6	107.9 ± 13.5
Abdominal obesity	828 (98.7%)	67.4 ± 13.0	30.8 ± 5.5	108.2 ± 13.2
Arterial hypertension	796 (94.9%)	67.9 ± 12.7	30.8 ± 5.7	108.0 ± 13.7
IFG	208 (24.8%)	63.6 ± 14.8	30.3 ± 5.2	106.6 ± 12.7
Diabetes mellitus type 2	491 (58.5%)	70.0 ± 11.1	31.2 ± 5.8	109.3 ± 13.9
TG	313 (37.3%)	63.8 ± 13.7	30.7 ± 4.7	108.0 ± 12.7
HDL	567 (67.6%)	68.1 ± 13.1	30.9 ± 5.9	108.0 ± 14.0

**Table 3:** Correlation between BMI and WC in the study group according to subject sex.

BMI/WC	*n*	*r*-correlation coefficient	*P*
Total	839	0.78	<0.05
Women	494	0.80	<0.05
Men	345	0.76	<0.05

**Table 4:** Characteristics of patients in the group with arterial hypertension and the group with normal arterial blood pressure.

Variables	Group with hypertension (*n* = 796)	Group with normotension (*n* = 43)	*P*
Age (years)	67.9 ± 12.7	60.7 ± 15.7	0.003
Body height (cm)	165.9 ± 8.6	168.7 ± 8.2	0.024
Body weight (kg)	85.0 ± 18.0	81.2 ± 12.7	0.167
BMI (kg/m^2^)	30.8 ± 5.7	28.4 ± 3.3	0.005
WC (cm)	108.0 ± 13.7	104.6 ± 10.2	0.143
MAP (mmHg)	125.9 ± 15.7	109.4 ± 10.6	0.000

**Table 5:** Correlation between BMI and waist circumference according to the presence of arterial hypertension or normotension.

BMI/WC	With arterial hypertension			With arterial normotension		
Total	*n* = 796	*r* = 0.79	*P* < 0.05	*n* = 43	*r* = 0.63	*P* < 0.05
Women	*n* = 471	*r* = 0.81	*P* < 0.05	*n* = 20	*r* = 0.55	*P* < 0.05
Men	*n* = 325	*r* = 0.76	*P* < 0.05	*n* = 23	*r* = 0.68	*P* < 0.05

**Table 6:** BMI to waist circumference ratio according to the presence of type 2 diabetes or no carbohydrate disorders.

BMI/WC	Diabetes mellitus type 2			No carbohydrate disorders		
Total	*n* = 491	*r* = 0.79	*P* < 0.05	*n* = 110	*r* = 0.76	*P* < 0.05
Women	*n* = 300	*r* = 0.81	*P* < 0.05	*n* = 66	*r* = 0.79	*P* < 0.05
Men	*n* = 191	*r* = 0.78	*P* < 0.05	*n* = 44	*r* = 0.71	*P* < 0.05

**Table 7:** Characteristics of patients from the study groups with and without diabetes mellitus type 2.

Variables	Group with type 2 diabetes (*n* = 491)	Group with no carbohydrate disorders (*n* = 110)	*P*
Age (years)	70.0 ± 11.1	63.9 ± 14.4	0.000
Body height (cm)	165.2 ± 8.3	167.1 ± 8.8	0.002
Body weight (kg)	85.5 ± 18.4	84.0 ± 16.8	0.329
BMI (kg/m^2^)	31.2 ± 5.8	30.0 ± 5.1	0.002
WC (cm)	109.3 ± 13.9	105.9 ± 12.7	0.001
MAP (mmHg)	124.4 ± 15.1	126.0 ± 17.0	0.460

**Table 8:** BMI to waist circumference ratio according to the presence or absence of IFG.

BMI/WC	IFG present			IFG absent		
Total	*n* = 208	*r* = 0.73	*P* < 0.05	*n* = 631	*r* = 0.80	*P* < 0.05
Women	*n* = 113	*r* = 0.78	*P* < 0.05	*n* = 381	*r* = 0.81	*P* < 0.05
Men	*n* = 95	*r* = 0.64	*P* < 0.05	*n* = 250	*r* = 0.79	*P* < 0.05

**Table 9:** Characteristics of patients from the study groups with IFG and without carbohydrate disorders.

Variables	Group with IFG (*n* = 208)	Group without IFG (*n* = 631)	*P*
Age (years)	63.6 ± 14.8	68.8 ± 12.0	0.000
Body height (cm)	167.1 ± 9.0	165.6 ± 8.4	0.036
Body weight (kg)	85.0 ± 16.4	84.8 ± 18.2	0.668
BMI (kg/m^2^)	30.3 ± 5.2	30.8 ± 5.7	0.498
WC (cm)	106.6 ± 12.7	108.3 ± 13.8	0.211
MAP (mmHg)	126.5 ± 17.0	124.5 ± 15.5	0.281

**Table 10:** BMI to waist circumference ratio according to the decreased levels of the HDL-cholesterol fraction or lack of lipid disorders.

BMI/WC	Decreased HDL-cholesterol levels			No lipid disorders		
Total	*n* = 567	*r* = 0.78	*P* < 0.05	*n* = 272	*r* = 0.79	*P* < 0.05
Women	*n* = 361	*r* = 0.80	*P* < 0.05	*n* = 133	*r* = 0.83	*P* < 0.05
Men	*n* = 206	*r* = 0.77	*P* < 0.05	*n* = 139	*r* = 0.73	*P* < 0.05

**Table 11:** Characteristics of patients from the study groups with decreased levels of the HDL-cholesterol fraction and without lipid disorders.

Variables	Group with decreased levels of the HDL-cholesterol fraction (*n* = 567)	Group without lipid disorders (*n* = 272)	*P*
Age (years)	68.1 ± 13.1	66.4 ± 12.5	0.021
Body height (cm)	165.6 ± 8.4	166.9 ± 8.8	0.035
Body weight (kg)	85.1 ± 18.7	84.2 ± 15.7	0.665
BMI (kg/m^2^)	30.9 ± 5.9	30.1 ± 4.7	0.110
WC (cm)	108.0 ± 14.0	107.6 ± 12.5	0.920
MAP (mmHg)	124.0 ± 15.5	127.3 ± 16.6	0.011

**Table 12:** BMI to waist circumference ratio according to the increased levels of triglycerides or lack of lipid disorders.

BMI/WC	Increased levels of triglycerides			No lipid disorders		
Total	*n* = 313	*r* = 0.76	*P* < 0.05	*n* = 526	*r* = 0.80	*P* < 0.05
Women	*n* = 151	*r* = 0.76	*P* < 0.05	*n* = 343	*r* = 0.83	*P* < 0.05
Men	*n* = 162	*r* = 0.77	*P* < 0.05	*n* = 183	*r* = 0.74	*P* < 0.05

**Table 13:** Characteristics of patients from the study groups with increased levels of triglycerides and without lipid disorders.

Variables	Group with increased TG levels (*n* = 313)	Group without lipid disorders (*n* = 526)	*P*
Age (years)	63.8 ± 13.7	69.7 ± 12.0	0.000
Body height (cm)	168.5 ± 9.0	164.6 ± 8.0	0.000
Body weight (kg)	87.5 ± 16.8	83.2 ± 18.1	0.000
BMI (kg/m^2^)	30.7 ± 4.7	30.7 ± 6.1	0.234
WC (cm)	108.0 ± 12.7	107.8 ± 14.0	0.496
MAP (mmHg)	124.9 ± 15.9	125.1 ± 16.0	0.955
